# Comparison of primary total hip replacements performed with a direct anterior approach versus the standard lateral approach: perioperative findings

**DOI:** 10.1007/s10195-012-0190-2

**Published:** 2012-04-05

**Authors:** Sanjay Meena

**Affiliations:** 1 Department of Orthopaedics, All India Institute of Medical Sciences (AIIMS), Ansari Nagar, New Delhi, 110029 India; 2 L-139, Sarita Vihar, New Delhi, 110076 India

Sir,

We read with interest the article entitled “Comparison of primary total hip replacements performed with a direct anterior approach versus the standard lateral approach: perioperative findings” by Alecci et al. [1] and congratulate the authors for this excellent study. However, there are some concerns:

- 1. The authors have not stated whether all the replacements were cemented, uncemented, or both, as minimally invasive surgery is more suited for uncemented [2].
- 2. The authors have also overlooked the difference in rehabilitation time after surgery or analgesic requirements between the two groups.

It would be helpful if the authors elaborated on the above-mentioned concerns.
